# Identification of Destruction Processes and Assessment of Deformations in Compressed Concrete Modified with Polypropylene Fibers Exposed to Fire Temperatures Using Acoustic Emission Signal Analysis, Numerical Analysis, and Digital Image Correlation

**DOI:** 10.3390/ma16206786

**Published:** 2023-10-20

**Authors:** Anna Adamczak-Bugno, Sebastian Lipiec, Jakub Adamczak, Josef Vičan, František Bahleda

**Affiliations:** 1 Faculty of Civil Engineering and Architecture, Kielce University of Technology, Av. 1000-An. of Polish State 7, 25-314 Kielce, Poland; jakubadamczak123@gmail.com; 2 Faculty of Mechatronics and Mechanical Engineering, Kielce University of Technology, Av. 1000-An. of Polish State 7, 25-314 Kielce, Poland; slipiec@tu.kielce.pl; 3 Faculty of Civil Engineering, University of Žilina, Univerzitná 8215/1, 010-26 Žilina, Slovakia; josef.vican@uniza.sk (J.V.); frantisek.bahleda@uniza.sk (F.B.)

**Keywords:** concrete, PP fibers, acoustic emission method, DIC system, fire conditions, FEM

## Abstract

This article presents the results of tests conducted to identify the failure process and evaluate the deformation of axially compressed concrete specimens modified with polypropylene fibers (PP). The test specimens were previously stored at ambient temperature and subjected to fire temperatures of 300 °C, 450 °C, and 600 °C. Acoustic emission (AE) signals were recorded during loading, along with force and strain measurements. The recorded AE signals were analyzed using the k-means clustering method. The compilation of the test results made it possible to determine the classes of signals characteristic of different stages of the material failure process and to indicate the differences in the failure mechanisms of specimens stored under ambient conditions and subjected to fire temperatures. Digital image correlation (DIC) measurements were conducted during the strength tests. A numerical model of the material (FEM) was also prepared, and a comparison of the obtained results was carried out.

## 1. Introduction

As is well known, it is possible to add polypropylene fibers to the concrete mix to ensure ductility, improve mechanical properties, and extend durability. This creates polypropylene-fiber-reinforced concrete (PPFRC). Thus, from a simple material, concrete becomes a complex solution that can be adapted to specific applications [1,2].

The requirements that concrete must meet relate not only to mechanical properties such as high compressive, tensile, and flexural strength but also to architectural qualities and safety. As more and more emphasis is now being placed on sustainability, reducing carbon dioxide production and extending the life of concrete structures is becoming one of the priority measures. Accordingly, efforts are being made to reduce the number and size of scratches and pores; reduce water permeability and chemical penetration; and increase strength, protect against corrosion, and reduce the negative effects of fire, freezing, thawing, impact, and abrasion. PPFRC is a material that can meet these requirements [3,4,5].

Polypropylene fibers (PPFs) are define by ASTM [6] as straight or deformed fragments of extruded, oriented, and cut polymer material. The main role PPFs play in the structure of concrete is to reduce scratching; not least to increase tensile and flexural strength [3,4].

The positive effect of the addition of fibers to concrete is also noticeable in tests of resistance to abrasion, impact, spalling, and freeze–thaw cycles. In addition, instead of the brittle type of failure, fiber concrete after scratching is able to carry loads with increasing crack width. On the other hand, lower porosity, permeability, and water absorption allow the extension of the life of the fiber concrete element [1,4,5,7].

Thanks to their scratch resistance, concretes with polypropylene fibers are used for the construction of, among other things [2],

- Sidewalks and pedestrian paths;
- Thin-walled façade elements;
- “Small architecture” elements;
- Repair layers of reinforced concrete elements (by the shotcrete method).

The chemical (corrosion) resistance of polypropylene fibers determines their use as an additive (supplementing structural reinforcement) also in the following concrete structures [5,7]:

- Monolithic water tanks;
- Settling tanks in sewage treatment plants;
- Sewage tunnels.

Polypropylene fibers are also a component of precast concrete tunnel lining elements as they counteract the explosive behavior of concrete and spalling at fire temperatures [4,5,7,8,9].

In construction practice, an important issue is the behavior of concrete during a fire. The dimensioning of load-bearing elements made of concrete and reinforced concrete with regard to the risk of fire is carried out, as a rule, on the basis of values tabulated in standards, taking into account the effects of fire on materials and elements. Concrete is considered a non-combustible and fire-resistant material. However, it is known that even under typical conditions of ordinary fires, serious damage can occur in it, the effects of which depend on the duration of the fire and the type of construction [8,9,10,11].

The decrease in the strength of concrete at temperatures around 200 °C is small; between 200 and 500 °C, the decrease in strength is much faster, and at 500 °C the basic compressive and tensile strength can decrease by several tens of percent. Above 500 °C, the strength of the concrete drops almost to zero [10,11,12,13,14].

The mechanical properties of fiber-reinforced concrete exposed to high temperatures have been the subject of many analyses. Sideris et al. [15] tested concrete reinforced with polypropylene fibers in the amount of 5 kg/[m^3^] in the temperature range of 100, 300, 500, and 700 °C. The samples were tested for compressive strength. It was found that the residual strength decreased almost linearly up to 700 °C. The influence of polypropylene fibers and silica fume on the mechanical properties of lightweight concrete exposed to high temperatures was investigated experimentally and statistically by Tanyildizi [16]. Mixtures containing silica fume (0% and 10%) and polypropylene fibers (0%, 0.5%, 1%, and 2%) were prepared. The compressive and bending strength of lightweight concrete samples was determined after being exposed to high temperatures (400, 600, and 800 °C). It has been shown that the compressive and flexural strength of lightweight concrete reinforced with polypropylene fibers decreases at temperatures above 400 °C. Poon et al. [17] reported that when exposed to a temperature of 600 °C, the relative compressive strength of plain Portland cement concretes increased slightly when 0.22% (*v*/*v*) PP fibers were added. At a temperature of 800 °C, the relative compressive strength was the same for concretes with and without PP fibers. Behnood and Ghandehari [18] and Hoff et al. [19] reported that the relative compressive strength of heated high-strength concretes containing polypropylene fibers was higher than that of concretes without PP fibers. Chen and Liu [20] found that the relative compressive strength of concretes containing polypropylene fibers was higher than that of concretes without PP fibers, for temperatures up to 600 °C. Komonen and Penttala [21] investigated the effect of high temperatures on the residual properties of Portland cement paste reinforced with polypropylene fibers exposed to temperatures up to 700 °C. Polypropylene fibers have been found to reduce compressive strength.

The research results show that the mechanism of polypropylene fibers, due to the fact that they melt at a temperature of 170 °C and create a porous structure, reduces the build-up of pore pressure inside the heated concrete structure [22,23,24,25]. They prevent spalling better than steel fibers, although they negatively affect other mechanical properties of concrete because they reduce the remaining compressive strength, elastic modulus, and tensile strength of the fired concrete [15,26]. Some of the conclusions regarding the use of PP fibers and their impact on the fire resistance of concrete or mortar are contradictory. For example, Xiao and Falkner [24] found that PP fibers increased the relative residual compressive strength and decreased the residual flexural strength of concrete. Other authors [27] found that the residual strength of concrete was not influenced by the addition of PP fibers exposed to temperatures in the range of 200–800 °C. Kalifa [28] reported that these fibers showed a beneficial effect on the three-layer strength of concrete when exposed to high temperatures.

NDT methods, especially acoustic emission (AE), are very widely used in condition monitoring of many engineering structures. Acoustic emission as a phenomenon can be defined as transient elastic waves resulting from local internal microdisplacements in the materials of the structures under test. The AE method has become a common NDT method used mainly for testing stationary equipment—tanks, pressure vessels, reactors, pipelines, bridges, etc. In these applications, the AE method is fully accepted and standardized. AE provides a wealth of information about the response of materials to applied stresses. It is useful for detecting and identifying increasing material defects. Thanks to its sensitivity, it can detect such processes as the formation and growth of microcracks, dislocation group displacement, cracking, sliding, or debonding of deposits [29,30].

The advantage of the AE method is the ability to examine the entire structure/element, locate any damage, and assess its threat to the structural integrity. AE application brings significant economic benefits [31,32,33,34].

In this article, through the use of multiparameter analysis of AE signals, the identification of destructive processes in PP-fiber-modified concrete during compression testing was carried out. Differences were indicated regarding the loading of specimens stored at room temperature and those subjected to fire temperatures. It was established that high temperatures led to a decrease in the mechanical parameters of the material, as well as a change in the mode of operation under load. It was confirmed that the acoustic emission method, due to the differences at low load values, can be a useful tool for evaluating the technical condition of PP-fiber-modified concrete after exposure to fire temperatures. So far, similar research directions have not been encountered.

In the present study, in addition to AE methods, numerical calculations using the finite element method and image analysis of the deforming specimen using a video extensometer (DIC methods) were used to evaluate the condition of PP-fiber-modified concrete elements after exposure to high temperatures. The use of the above-mentioned diversified test methods will allow a comprehensive approach to the evaluation of the behavior of the analyzed material [35,36,37,38,39,40]. In particular, in the current literature, there are no studies where differentiated test methods were implemented in fire tests of polypropylene-fiber-modified concrete. The results obtained can be helpful for specific engineering and implementation concepts of elements made of the tested concrete under operating conditions in a wide temperature range.

## 2. Materials and Methods

### 2.1. Materials

Concrete cubes of 150 × 150 × 150 mm were tested. The pieces had a concrete structure with the addition of dispersed PP fibers. The test material was obtained from an entity offering to sell modified concrete for industrial use. It was concrete on Portland cement with 10% fiber content. The overall density was 2417 kg/m^3^. The length of the fibers was 12 mm, and the diameter was 29 μm. Information on the exact composition of the material was not obtained, as the formula is protected by patent law.

Four series of three samples each were tested. Samples from the first group, A, were stored at ambient room temperature (±20 °C). Samples from the second test series, B, were subjected to firing in a laboratory furnace. The processing temperature was 300 °C. The annealing time from the time the set temperature was reached was 3 h. Samples from the third test series, C, were subjected to firing in a laboratory oven at 450 °C. The annealing time from the time the set temperature was reached was 3 h. Samples from the fourth test series, D, were subjected to firing in a laboratory furnace at 600 °C. The annealing time from the time the set temperature was reached was 3 h.

The firing time of the specimens in the furnace was set to bring the temperature to a uniform value throughout the volume of the conditioned parts. Samples cooled under air conditions at ambient room temperature were tested.

### 2.2. Methods

**Figure 1:**
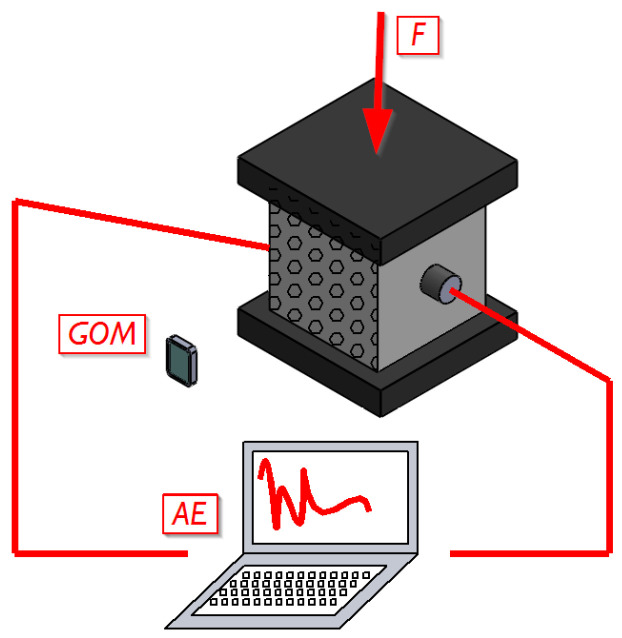
Scheme of test methods included in the compression tests of specimens made from PP-fiber-modified concrete.

The cubic specimens were subjected to axial compression using a Zwick Roell testing machine. A schematic diagram and view of the test stand are shown in Figure 1. The compression speed was 0.1 MPa/s.

Acoustic emission signal acquisition was carried out during the study. For this purpose, an AEWin acoustic emission processor (Mistras, Princeton Junction, NJ, USA) and two Vallen sensors of the VS75-SIC-40dB type were used.

During the tests, image recording was conducted on the front of the specimen for later analysis using DIC–GOM Suite software v2022, rev. 157716.

#### 2.2.1. Acoustic Emission Method

The acoustic emission (AE) method belongs to the group of passive methods, that is, the AE apparatus does not emit signals and does not affect the physical state of the object/element under test but only records physical effects spontaneously arising in the monitored object. The sources of the acoustic emission signal are the formation and propagation of microcracks, corrosion processes, the cracking of strings in prestressed structures, the escape of gas through leaks in the pressure structure, or damage to the insulation of high-voltage power equipment, that is, processes in which elastic waves are formed and propagate in the object. The AE apparatus records the signal generated in the tested object during its normal operation or during tests. The elastic waves generated in the AE source propagate from the source in all directions in the volume of the monitored object. The elastic waves reach the AE sensor and are then transmitted to the AE analyzer in the form of electrical voltage changes. The mathematical model of the propagation of these waves and the accompanying changes in their form is quite complex and uses the properties of the Green’s function [34,41,42].

The measurement apparatus consists of AE sensors that convert AE signals into an alternating electrical voltage, AE analyzers that amplify this voltage and eliminate signals that do not originate from the monitored source (acoustic background), and an AE signal recorder [36,42,43].

For the classification of AE signals, NOESIS v12 software is very often used, which is mainly based on the pattern recognition method, in two versions: an arbitrary class division (unsupervised), USPR, and a self-learning one, in which the class division is performed using pattern signals (supervised), SPR [34,36,41,42,43].

In the first case, arbitrary pattern analysis is mainly used when creating a database of pattern signals if the number of classes is unknown. The second method is used, in cases where pattern signals characterizing a given destructive process are available. The benchmark signals are signals previously collected in a database, generated during independent experiments [36,42].

For statistical methods used for object recognition, an important consideration is the optimal choice of acoustic emission parameters to be recorded. Many acoustic emission parameters have a strong correlation with each other, so that they can carry the same information about the AE source. The degree of correlation between AE parameters is determined by so-called dendrograms [32,33].

An important issue that may affect the accuracy of the calculations, which should also be taken into account, is the number of iterations needed to obtain satisfactory results. It has been accepted that a sufficient number of iterations is 10,000. Decreasing this number results in a significant decrease in the fit of the signals in each class, while increasing it slows down the analysis process, and the obtained results improve the fit only slightly [31,32,33,34].

The NOESIS program uses different clustering methods, and the manual does not provide guidance on which one to choose. In the discussed analyses, the Fuzzy K-means algorithm was used [29,30].

The k-means method is a method belonging to the group of cluster analysis algorithms, i.e., an analysis involving the search for and separation of groups of similar objects (clusters). It represents a group of non-hierarchical algorithms. The main difference between non-hierarchical and hierarchical algorithms is the need to specify the number of clusters in advance [29,30,31,32,33,34].

Using the k-means method, k different, possibly distinct clusters are created. The algorithm involves moving objects from cluster to cluster until intra-cluster and inter-cluster variability is optimized. The similarity within a cluster should be as high as possible, while the separate clusters should differ from each other as much as possible [34,41].

The methodology of the algorithm can be described as follows [34,36,41,42,43]:

1. Determining the number of clusters.

One method of determining the number of clusters is to contractually select it and possibly change this number later to obtain better results. The choice of the number of clusters may also be based on the results of other analyses.

2. Determination of initial cluster means.

The cluster means, the so-called centroids, can be selected in several ways: random selection of k observations, selection of the first k observations, or selection in such a way as to maximize the cluster distances. One of the most common methods is to run the algorithm several times and select the best model when the initial cluster means were selected randomly.

3. Calculation of the distance of objects from the cluster centers.

The choice of metric is a very important step in the algorithm. It influences which observations will be considered similar and which will be considered too different from each other. The most commonly used distance is the Euclidean distance (used to divide the signals analyzed in the paper). The square of this distance or the Chebyshev distance is also used.

4. Assignment of objects to clusters.

For a given observation, compare the distances to all clusters and assign it to the cluster to which it has the closest center.

5. Determination of new cluster centers.

Most often, a new cluster center is a point whose coordinates are the arithmetic mean of the coordinates of the points belonging to a given cluster.

6. Repeat steps 3, 4, and 5 until a stop condition is met.

The most commonly used stop condition is the number of iterations set at the beginning or the absence of object transfers between clusters.

#### 2.2.2. Numerical Analysis

**Figure 2:**
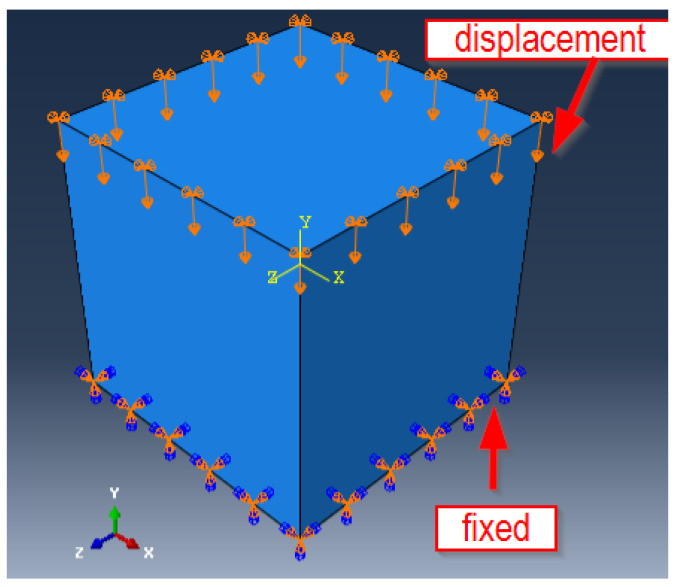
Numerical model of a compression specimen of concrete with PP fiber modification.

The results of the experimental tests served as the basis for the numerical calculations. Numerical models were developed for specimens identical to those used in the experimental tests. These were 150 × 150 × 150 mm cubes made of PP-fiber-modified concrete. The lower surface of the specimen was assumed to be fixed in the boundary conditions, while the displacement of the upper wall was applied (as shown in Figure 2). The displacement assumed in the numerical calculation program corresponded to the moment of occurrence of the maximum force during the compression tests of the specimens. An eight-node, three-dimensional finite element type C3D8R was used in the model [44]. The compression specimen model contained 8000 finite elements. The size of the smallest finite element was 7.89 × 7.89 mm. A material model in the form of the concrete plasticity model (CDP) was used in the numerical calculation program. Details relating to the material model used are shown in Table 1. The definition of the material model in the numerical calculations used the guidance provided in the papers [36,45,46,47,48,49,50,51,52]. This involved the definition of the stress–strain curve obtained in compression and tension tests of the material under analysis. An important aspect when carrying out numerical analyses is the possibility of verifying the results obtained, e.g., by comparing them with experimental results or with the results obtained from the analysis of real-time laboratory tests. For this reason, a videorejection of the specimen deformation process (DIC) was used in the experimental program. After the numerical model was developed, the modeled specimens were simulation-loaded with values identical to the experimental values. At characteristic moments (e.g., maximum test or failure force), the stress and strain values were read off in the inner regions of the specimens. The aim of this was to carry out the analysis according to a hybrid method of analysis: complementing the experimental results with numerical results and analyses using DIC to determine the quantities characterizing the failure process. Modeling of the specimens, simulation loading, and calculations were carried out using Abaqus ver. 6.12-2 [53].

#### 2.2.3. Digital Image Correlation

The Digital Image Correlation Method (DIC) enables the measurement of in-plane and out-of-plane displacements and the calculation of object deformations resulting from the load of external forces and environmental forces (changes in humidity, temperature, pressure of the environment in which it is located object). The measurement involves taking a series of photos before and after loading the tested object. The surface of the tested object must have a certain random texture (spot structure). Typically, when measuring engineering objects, such a texture should be applied to the object before starting the measurements, which is not acceptable in most cases of works of art; it may be, for example, the natural texture of a painting, in the form of intense color differentiation and/or a clear brush stroke. One of the images in the series is selected as the reference image for all subsequent analyses. The reference image is divided into small rectangular regions, called subsets. The DIC algorithm tracks the position of each subset from the reference image in all other images of the measurement series. Finding subsets is performed by calculating the cross-correlation coefficient. For each subset, in-plane displacement vectors (*u* and *v*) are calculated, and out-of-plane displacements (*w*) are determined using triangulation. By using interpolation methods, sub-pixel accuracy is achieved. The output data are a set of displacement maps, which can then be used to calculate strain maps in the (*ε_xx_*,*ε_yy_*) plane [54,55,56,57]. During the laboratory tests, a phone with camera parameters was used to record images of the deforming specimen: 50 Mpx sensor, 0.64 mm pixel size, 1/2.76 inch sensor size, 1920/1080 video resolution, ƒ/1.8.

## 3. Results

### 3.1. Experimental Studies

**Figure 3:**
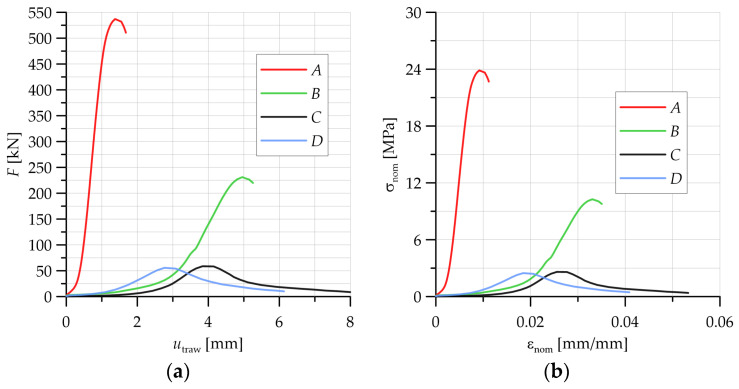
Experimental results of representative specimens: (**a**) force–displacement relationships and (**b**) nominal stress–strain relationships.

In the laboratory testing program, compression tests were carried out on 150 × 150 mm cubes made of concrete with PP fiber reinforcement. The tests were carried out on a Zwick testing machine equipped with automated control and data recording systems. The specimens were loaded to failure. Figure 3a shows the force–displacement diagrams recorded during uniaxial compression tests on the specimens. Based on the data recorded during the tests, nominal (engineering) stress–strain relationships of the material were developed (Figure 3b).

**Figure 4:**
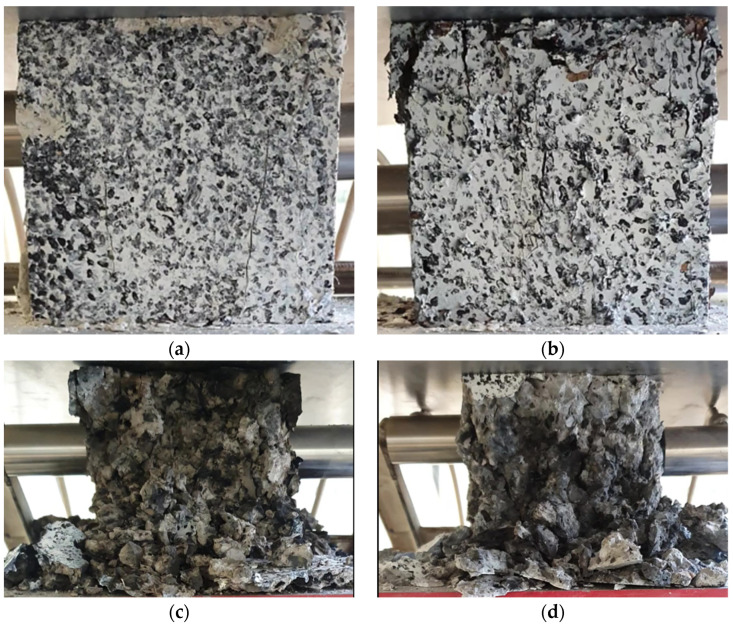
View of samples on the testing machine after the compression test, variant of heating the material: (**a**) A, (**b**) B, (**c**) C, and (**d**) D.

As the annealing temperature of the test specimens increased, there was a decrease in the nominal stress level but an increase in the strain at failure. In particular, a decrease in the maximum nominal stress level was clearly visible from an annealing temperature of 450 °C (material designated C). When observing the state of the specimens after the compression test for the specimen in the initial state (A) and for the annealing temperature of 300 °C (material B), there were fractures on the observed specimen front, but there was no clear shattering of the specimens (Figure 4a,b). Significant failure of the specimens after the compression test occurred for annealing temperatures of 450 °C (material C) and 600 °C; Figure 4c,d show shattering of the specimens into multiple pieces. The details and observations made during the compression test of the accepted specimens formed the basis for the development of the numerical models in the subsequent strength analyses.

### 3.2. Results of Tests Using the Acoustic Emission Method

Fourteen AE signal parameters were used to create a reference signal database of destructive processes in PP-fiber-modified concrete specimens:

1. Duration (μs);
2. Rise time (μs);
3. Decay angle (rad);
4. RMS (V);
5. Counts;
6. Counts to peak;
7. Amplitude (mV);
8. Energy (EC);
9. Average frequency (kHz);
10. Reverberation frequency (kHz);
11. Initiation frequency (kHz);
12. Absolut energy (aJ);
13. Signal strength (pVs);
14. Average signal level (dB).

The measure of the matching of signals to individual classes was the R^2^ coefficient, which in each case was not less than 0.9. The evolution of the crack sequence was divided into seven stages based on microcrack and macrocrack levels. 

The processes were assigned to groups of acoustic emission signals isolated using the *K-means* algorithm:

- Linear elastic deformation—class 1 (red square);
- Microcrack initiation—class 2 (blue rhombus);
- Growth of microcracks—class 4 (black lower triangle);
- Coalescence of microcracks—class 5 (yellow upper triangle);
- Formation of macrocracks—class 3 (green dot);
- Macrocracks–crack coalescence—class 6 (gray plus);
- Failure—class 7 (x in pistachio color).

Table 2 shows the ranges of some acoustic emission parameters for individual grouping classes.

**Figure 5:**
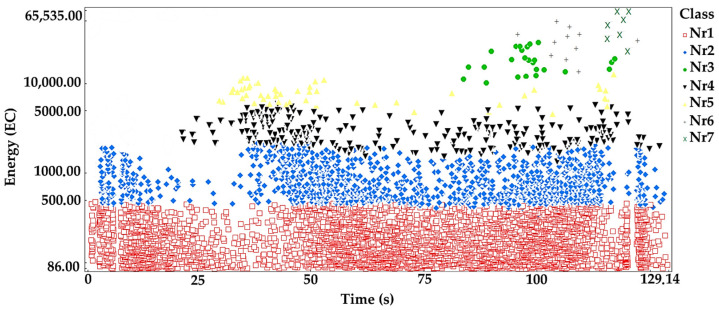
Distribution of individual classes of AE signals for the energy parameter for one of the samples from the A series.

Analyzing the graph of the energy distribution of acoustic emission signals of individual classes over time for an exemplary sample from series A (Figure 5), it can be concluded that in the time course from the beginning of the loading process, there were signals corresponding to the elastic work of the material. Shortly after applying the load, signals related to the beginning of the development of microcracks appeared. The increase in load led to their gradual growth and coalescence. The occurrence of damage on a macroscale quickly led to the destruction of the element. Subsequent processes were related to the occurrence of signals of increasingly higher energy.

**Figure 6:**
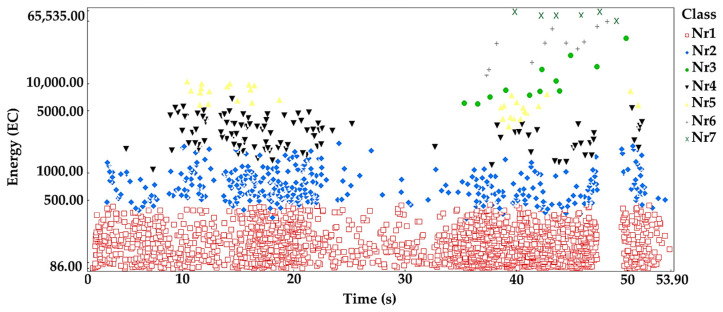
Distribution of individual classes of AE signals for the energy parameter for one of the samples from the B series.

Analyzing the graph of the energy distribution of acoustic emission signals of individual classes over time for an exemplary sample from series B (Figure 6), it can be concluded that in the time course from the beginning of the loading process, there were signals corresponding to the elastic work of the material. Shortly after applying the load, signals appeared related to the beginning of the development of microcracks. The increase in load led to their gradual growth and coalescence. The occurrence of damage on a macroscale quickly led to the destruction of the element. It was noted that for the samples from the series in question, there was a moment when there was a break in the development and coalescence of microcracks. Subsequent processes were related to the occurrence of signals of increasingly higher energy.

**Figure 7:**
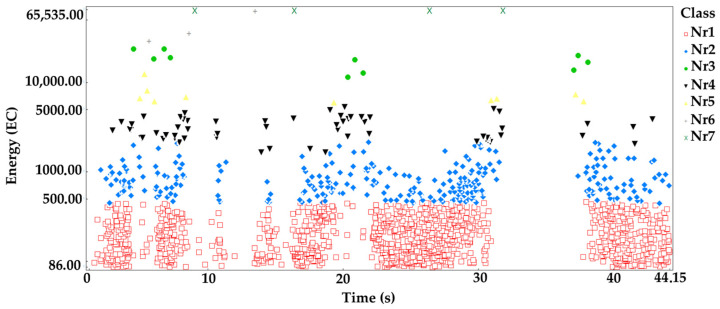
Distribution of individual classes of AE signals for the energy parameter for one of the samples from the C series.

Analyzing the graph of the energy distribution of acoustic emission signals of individual classes over time for an exemplary sample from series C (Figure 7), it can be concluded that signals of all classes appeared in the time course from the beginning of the loading process. The process of destruction of the material was much faster than in the case of samples in an air-dry state and fired at a temperature of 300 °C. The destruction occurred in stages. Subsequent processes were related to the occurrence of signals of increasingly higher energy.

**Figure 8:**
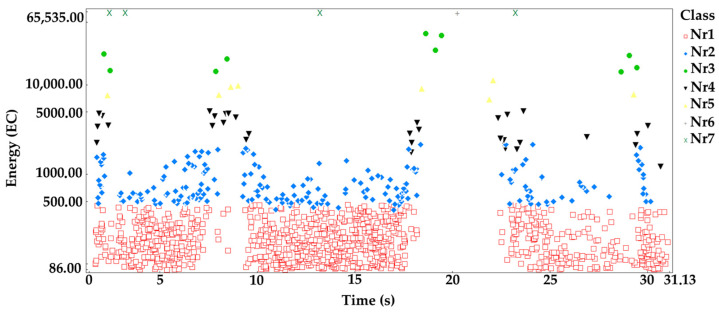
Distribution of individual classes of AE signals for the energy parameter for one of the samples from the D series.

Analyzing the graph of the energy distribution of acoustic emission signals of individual classes over time for an exemplary sample from series D (Figure 8), it can be concluded that signals of all classes appeared in the time course from the beginning of the loading process. The process of destruction of the material was much faster than in the case of samples in an air-dry state and fired at a temperature of 300 °C. The destruction occurred in stages. Subsequent processes were related to the occurrence of signals of increasingly higher energy.

### 3.3. Results of Numerical Calculations and DIC

As a result of the performed numerical calculations, the values of stresses and strains were determined in simulation-loaded specimens identical to those used in the laboratory tests. The numerical model assumed a displacement of the upper wall of the specimen, the value of which corresponded to the displacement recorded when the maximum force was reached in compression tests. The numerically determined maximum Mises effective stress values and the component of the stress tensor in the compression direction, as well as strains, both effective and in the direction of the compressive force, are shown in Table 3.

**Figure 9:**
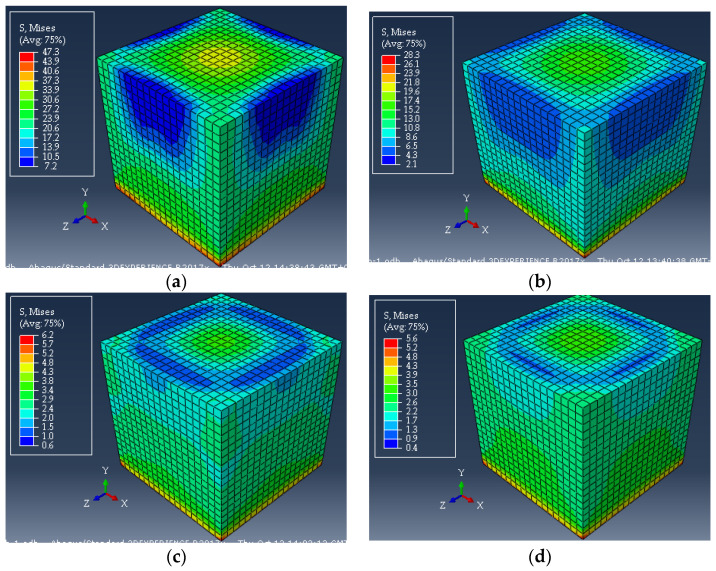
Numerically determined Mises stress maps for specimens with material designation: (**a**) A, without firing; (**b**) B, 300 °C; (**c**) C, 450 °C; and (**d**) D, 600 °C.

The numerically determined levels of effective stress and stress in the direction of the compressive force decreased with increasing temperature at which the PP-fiber-modified concrete cubes were fired. For the specimen without firing, the maximum stress level oscillated around 50 MPa. When the heat was applied to the sample at 300 °C, the stress level was reduced to a value of approximately 30 MPa. A significant decrease in stress was recorded from the firing temperature of the samples of 450 °C. At that point, the stresses were at a level of 6–7 MPa. This is almost an eightfold reduction in stresses with respect to the specimen without heat treatment. As the firing temperature of the PP-fiber-modified concrete increased, the level of numerically determined deformations increased. A notable exception in the analyzed specimens is the case of the concrete cube firing at 300 °C, where, as could be seen from the force–displacement diagram of the crosshead, the maximum force occurred at a higher level of deformation than occurred at the firing temperatures of the analyzed concrete: 450 °C and 600 °C. The contribution of the modifying polypropylene fiber in the firing tests showed a decrease in stress levels with increasing temperature, while an increase in strain values was shown. This was due, among other things, to the contribution of the modifying additive and its role in melting into the concrete at high firing temperatures. This was the result of an increase in the plasticity characteristics of the concrete with the modifying fibers. These results may be of great importance for the planned operation, especially fire operation, of concrete with the analyzed share of PP modifying fibers. Maps of the Mises effective stress distributions for the four analyzed specimens are shown in Figure 9.

**Figure 10:**
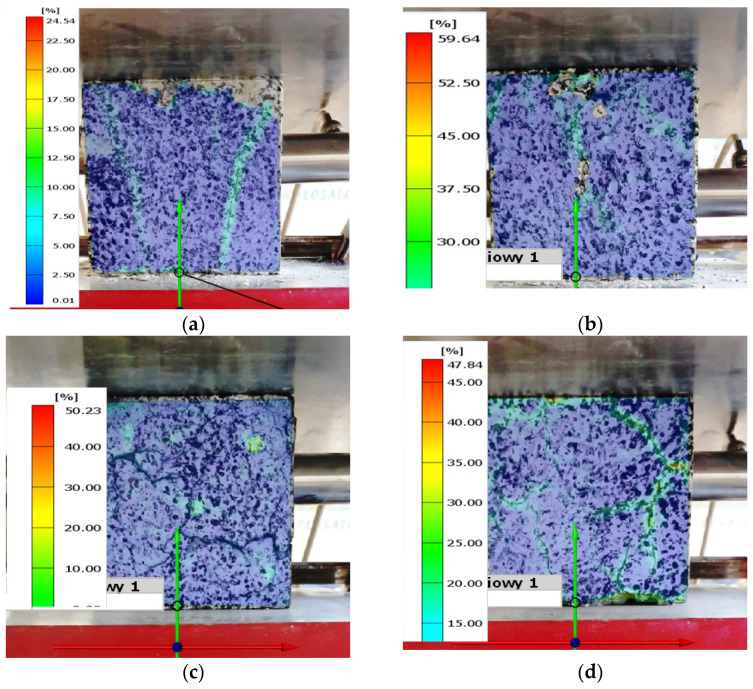
Values of effective strain obtained by DIC analysis for PP-fiber-modified concrete specimens for firing temperatures (for the moment of maximum force): (**a**) A, without firing; (**b**) B, 300 °C; (**c**) C, 450 °C; and (**d**) D, 600 °C.

The laboratory tests were accompanied by image recording of the deformed specimens in order to determine the distribution of effective deformations in the material using GOM software v2022, rev. 157716 (presented in Figure 10). These results were compared with the results of the numerical calculations. The maximum difference was recorded for a concrete firing temperature of 300 °C of within 5%. For all specimens, the verification of the results of the numerical calculation should be regarded as positive. This is of great importance, particularly when analyzing the strength of non-standard materials, such as PP-fiber-reinforced concrete, which is subjected to firing tests over a wide temperature range.

## 4. Discussion

Based on the analyses of the research results presented above regarding the destruction process of compressed concrete samples stored at ambient temperature and subjected to the influence of fire temperatures, performed using the analysis of AE signals, numerical analysis, and DIC, certain generalizations were formulated.

- When loading axially compressed concrete samples modified with PP fibers, various mechanisms (processes) of material destruction occurred: linear elastic deformation, microcrack initiation, microcrack growth, microcrack coalescence, macrocrack formation, macrocrack coalescence, and failure.
- The destructive processes were analogous for samples stored at ambient temperature and those exposed to fire temperatures.
- The development of destructive processes in the material was associated with the emission of signals with increasingly higher energy levels.
- The destruction of samples stored at ambient temperatures and those exposed to fire temperatures involved the emission of signals with similar energy.
- In the case of samples stored at ambient temperature conditions and subjected to fire temperatures of 300 °C (series A and B), the processes occurred sequentially until the components failed. The course of individual classes of acoustic emission signals was close to linear.
- In the case of samples subjected to fire temperatures of 300 °C (series B), a break in the development and coalescence of microcracks was noted. This fact was associated with the possibility of local strengthening of the material due to the increase in the degree of compactness of the material structure by the melted plastic (PP fibers).
- In the case of samples subjected to fire temperatures of 450 °C and 600 °C (series C and D), a significantly smaller number of events and a change in their distribution over time were found.
- In the case of samples subjected to fire temperatures of 450 °C and 600 °C (series C and D), it was found that all destructive processes occurred in steps at certain time intervals. This fact was associated with the increase in the degree of porosity of the material as a result of high temperatures.
- Through the use of numerical calculations using the finite element method, it was possible to determine the values of stresses and strains in the analyzed specimens from concrete with PP fiber reinforcements.
- The validity of the numerical results obtained in terms of deformation values was verified by comparing the numerical results and those obtained by analyzing the deforming image of the specimen (DIC).

## 5. Conclusions

Based on the conducted research and analyses, the following conclusions were drawn:

- Fire temperatures of 300 °C, 450 °C, and 600 °C reduced the mechanical strength of concrete modified with PP fibers.
- As the temperature at which the specimen was fired increased, there was a significant decrease in the numerically determined levels of effective stress and the component of the stress tensor in the direction of the loading force. On the other hand, the level of maximum strain increased.
- Good agreement was obtained between the deformations obtained by numerical calculations and those determined by DIC analysis.
- Compression of concrete samples modified with PP fibers was associated with the emission of acoustic signals characteristic of various destructive processes occurring in the material.
- Fire temperatures influenced the change in the number and distribution of acoustic emission signals of individual classes generated during compression of concrete samples modified with PP fibers.
- Differences in the number and distribution of acoustic emission signals of individual classes occurred at low levels of load and deformation.
- The analysis of acoustic emission signals according to the energy parameter over time allowed for conclusions about the degree of advancement of the destructive processes taking place in the material structure.
- The acoustic emission method can be fully useful for monitoring the condition of fiber-modified concrete elements or structures under load. This applies to both materials operated at ambient temperatures and those exposed to fire temperatures.

## Figures and Tables

**Table 1:** Details of the CDP material model used in the numerical model.

Material Characterics/Specimen	A	B	C	D
*E*, GPa	24	16	8	7
ν	0.2	0.2	0.2	0.2
Dilation angle	30	30	30	30
Eccentricity	0.10	0.10	0.10	0.10
*f*_b0_/*f*_c0_ (i.e., *σ*_b0_/*σ*_c0_)	1.16	1.16	1.16	1.16
*K*	0.67	0.67	0.67	0.67
Viscosity parameter	0.00	0.00	0.00	0.00
*σ*_cu,_ MPa	23.87	10.08	2.60	2.47
*σ*_tu,_ MPa	5.12	3.47	1.44	1.40

**Table 2:** Ranges of AE signal characteristics for grouping classes.

Class	1	2	3	4	5	6	7
Signal strength (pV∙s)	5.40 × 10^5^– 2.92 × 10^6^	1.97 × 10^6^– 1.35 × 10^7^	8.17 × 10^7^– 2.03 × 10^8^	3.60 × 10^7^– 8.41 × 10^7^	3.60 × 10^7^– 4.38 × 10^5^	1.20 × 10^8^– 4.26 × 10^8^	5.03 × 10^8^– 8.63 × 10^9^
Max amplitude (V)	88	94	95	96	96	96	97
Frequency [kHz]	12–87	13–70	24–54	15–58	22–56	3–52	0–35
Max energy (eu)	445	2167	32,473	6812	13,475	65,535	61,535
Max duration (µs)	18,679	57,367	419,761	101,569	169,780	925,479	1 × 10^7^

**Table 3:** Results of numerical calculations.

Specimen Symbol	Mises Stress *σ*_eff_ [MPa]	Stress in the Compression Direction *σ*_22_ [MPa]	Effective Strain *ε*_eff_ [mm/mm]	Strain in the Compression Direction *ε*_22_ [mm/mm]
A	47.28	50.38	0.24	0.25
B	28.32	30.45	0.63	0.64
C	6.16	6.69	0.52	0.59
D	5.65	6.10	0.49	0.50

## Abbreviations

Duration (μs)	The time between the hit start and the hit end sample points.
Rise time (μs)	The time from hit start to the max amplitude sample point.
Decay angle (rad)	The adjusted angle of the straight line connecting the threshold level (in volts) at the hit end time with the maximum signal amplitude (at pre-amp output) point to the horizontal.
RMS (V)	A statistical measure defined as the square root of the mean of the squares of the AE hits amplitude. The RMS of the AE hits amplitude is usually used because it both is unambiguous and has physical significance. RMS is usually measured on a linear scale and reported in volts, similar to the amplitude units.
Counts	The integer part of the sum of the positive and negative signal threshold crossings plus 1 over 2.
Counts to peak	The integer part of the sum of the positive and negative signal threshold crossings from hit start to maximum amplitude plus 1 over 2.
Amplitude (mV)	The maximum amplitude of the signal at the sensor converted to dBae.
Energy (EC)	The adjusted area under the voltage curve computed at the sensor. The physical unit is μVs, but it is not used to describe the number, as this is an adjusted energy measurement.
Average frequency (kHz)	The signal counts over the signal duration in μsec.
Reverberation frequency (kHz)	The signal counts from maximum amplitude to hit end over the time from maximum amplitude to hit end in μsec.
Initiation frequency (kHz)	The signal counts to peak over the signal rise time in μsec.
Absolut energy (aJ)	The true energy of the signal on a 10 kOhm resistor computed at the sensor.
Signal strength (pVs)	The area under the voltage curve computed at the sensor.
Average signal level (dB)	A statistical measure defined as the average of the AE signal amplitude. Because the ASL considers the AE amplitude in a logarithmic scale, it must be reported in dB units.
*E* (Pa)	Young’s modulus.
ε_ij_ (mm/mm)	The individual components of the strain tensor.
ε_eff_ (mm/mm)	Effective strain.
Ν	(Dimensionless factor)—Poisson’s ratio.
σ_ij_ (Pa)	The individual components of the stress tensor.
σ_eff_ (Pa)	Mises effective stresses.
σ_cu_ (Pa)	Maximum compressive stress of concrete.
σ_tu_ (Pa)	Maximum tensile stress of concrete.
